# AI based medical imagery diagnosis for COVID-19 disease examination and remedy

**DOI:** 10.1038/s41598-024-84644-1

**Published:** 2025-01-10

**Authors:** Ashraf Aboshosha

**Affiliations:** https://ror.org/04hd0yz67 grid.429648.5 0000 0000 9052 0245 Rad. Eng. Dept., National Center for Radiation Research and Technology (NCRRT), Egyptian Atomic Energy Authority (EAEA), Cairo, Egypt

**Keywords:** AI, COVID-19, AI-based medical diagnosis, AI-remedy, Innate immunity, Cross-symptoms, Biological techniques, Computational biology and bioinformatics, Ecology, Immunology, Systems biology

## Abstract

COVID-19, caused by the SARS-CoV-2 coronavirus, has spread to more than 200 countries, affecting millions, costing billions, and claiming nearly 2 million lives since late 2019. This highly contagious disease can easily overwhelm healthcare systems if not managed promptly. The current diagnostic method, Molecular diagnosis, is slow and has low sensitivity. CXR, an initial imaging tool, provides rapid results, but is less sensitive compared to CT scans. This article focuses on using AI for two main objectives: classifying the severity of COVID-19 and determining the appropriate treatment. Highlights key factors in the diagnosis and treatment of COVID-19, addressing questions such as: 1. For COVID-19 is innate immunity more important or acquired immunity? 2. Is the COVID-19 an immunity disorder or Acute Respiratory Distress Syndrome(ARDS)? 3. Is the cross mortality due to aging more dangerous than COVID-19? 4. Is COVID-19 a seasonal disease due to the deficiency of vitamin D in winter? 5. Is it better to treat COVID-19 as an epidemic or a pandemic?

## Introduction

**Figure 1:**
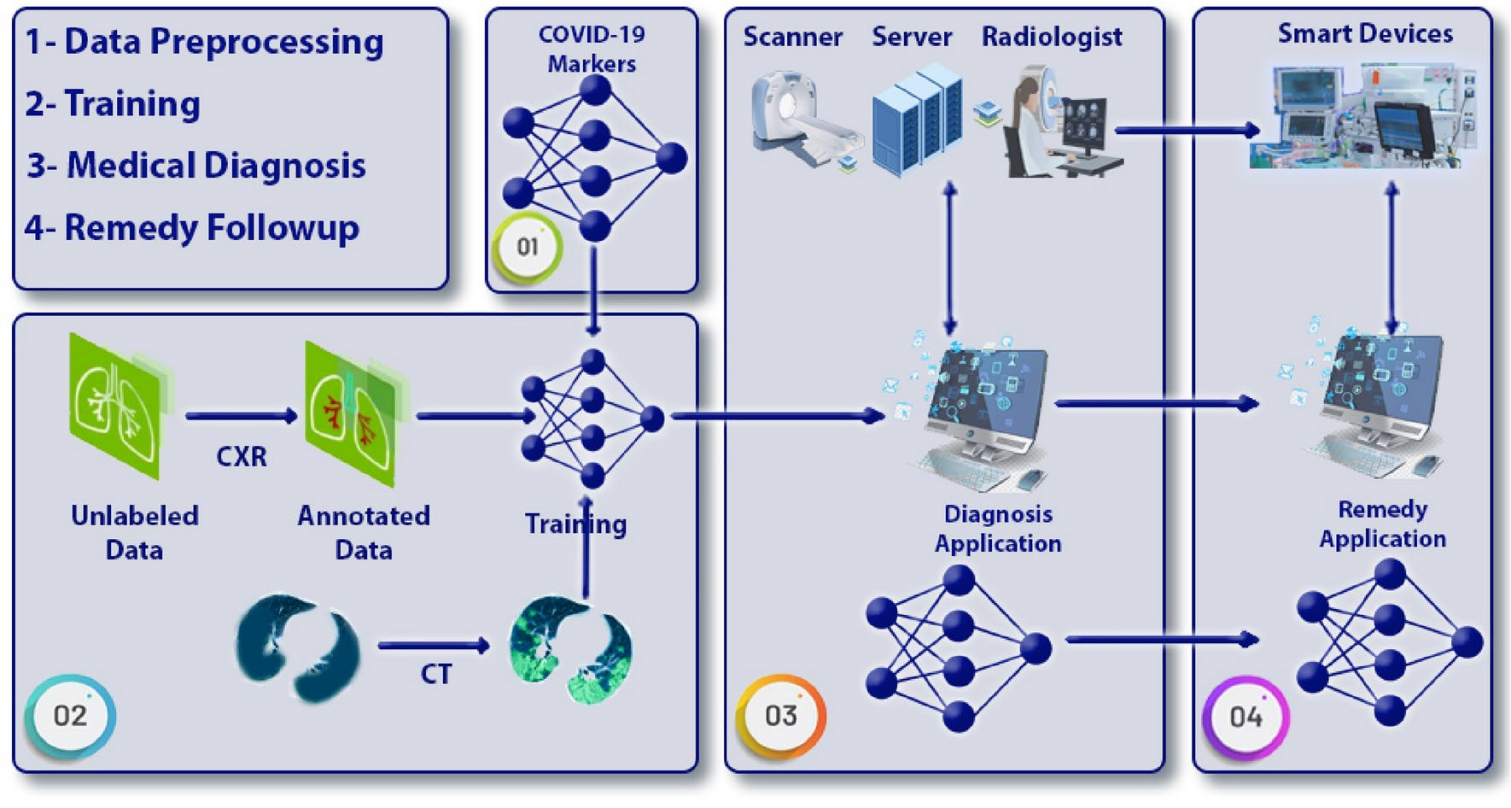
DL based COVID-19 diagnosis and remedy.

The COVID-19 outbreak began in late 2019 and caused a global uproar. Although most patients had mild-moderate symptoms such as cough, cold, myalgia, sore throat, muscle pain, nausea, loss of taste/smell and headaches, others faced severe conditions like acute respiratory distress syndrome (ARDS), severe hypoxia, and multi-organ failure, leading to fatalities. Currently, the virus continues to spread and new mutations are emerging. Patients with COVID-19 may experience a cytokine storm, marked by a significant release of cytokines such as IL-6 and IL-1, resulting in the immune system attacking itself and contributing to fatalities among Sars-Cov-2 patients, see Fig. 1.

**Figure 2:**
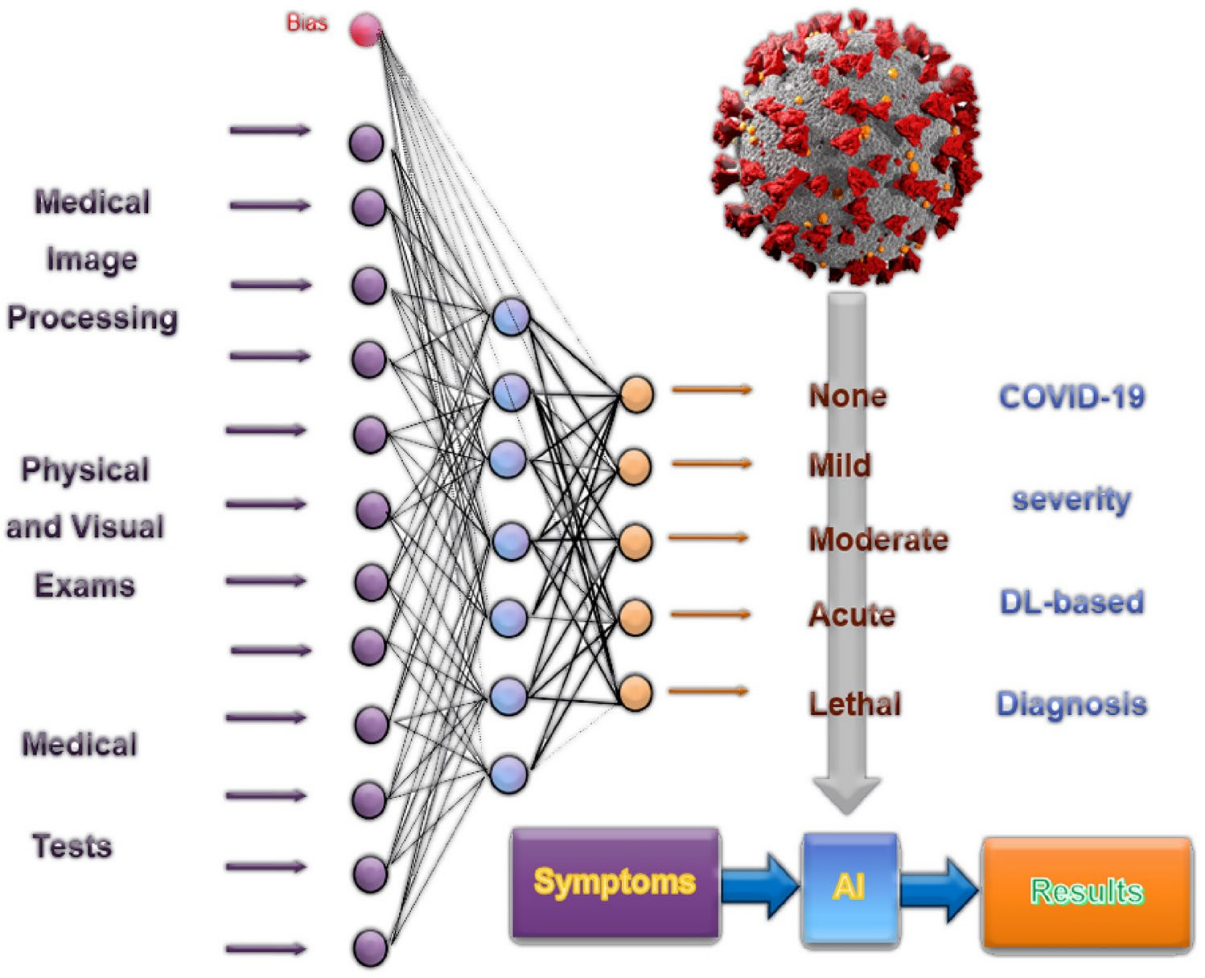
DL implementation of COVID-19 AI based diagnosis.

The severe symptoms of COVID-19 have lessened since the introduction of vaccines. Nevertheless, certain COVID-19 patients remain susceptible to severe outcomes. Older patients and people with comorbidities such as hypertension, diabetes, cancer, etc. are still at risk. Early identification of these patients is essential to offer timely medications and treatments, preventing avoidable deaths. Specific drugs have been developed and proven effective in averting severe COVID-19 symptoms. Administering these drugs in the early stages of the disease is crucial for their efficacy. Applications of artificial intelligence (AI) in healthcare have been widespread. Diagnostic and prognostic models, decision support systems, and predictive modeling are being created to help healthcare professionals through machine learning (ML). These technologies are also playing a role in the fight against COVID-19. General Artificial Intelligence (GAI) improves the transparency and comprehensibility of models. GAI can visually depict the rationale behind a patient’s prediction. Furthermore, GAI has found applications in finance, engineering, pharmacy, medicine, and commerce, see Fig. 2. The data are partition into subsets as in Fig. 2. It is a direct form of cross-validation which inherits several advantages, Bias-Variance Tradeoff, model selection, and parameter tuning while bootstrapping is inappropriate with these data. To improve the DL performance, ADAM optimization has been applied.

Several markers like C-reactive protein (CRP), D-Dimer, lactate dehydrogenase (LDH), neutrophil to lymphocyte ratio (NLR), and Ferritin exhibit significant changes prior to the manifestation of severe symptoms. Using these markers, machine learning models can be used to forecast the severity of COVID-19 beforehand. Identifying patients with unfavorable prognoses in a timely manner and establishing straightforward and reliable prediction methods in everyday clinical settings are essential to provide optimal treatment in healthcare facilities. COVID-19 is a pandemic throughout the whole world, but this research work recommends dealing with it as an epidemic in each country independently where weather, geographic location, religious traditions, environment, culture, local nutrition, skin color and race affect immunity immensely.

AI has emerged as a promising tool, providing data-driven solutions to tackle the pandemic. Advanced machine learning and deep learning techniques can unlock insights using large-scale data sets related to coronavirus transmission, disease progression, patient outcomes, population mobility, and health care operations. AI-based predictive analytics, intelligent diagnosis, risk assessment systems, decision support platforms, and computational drug discovery have great potential to improve COVID-19 prediction, detection, control, and treatment. However, to have a real impact on clinical and public health, a nuanced understanding of the relevant epidemiological, clinical, ethical and social contexts is essential. The power of approximation is the most important feature of DL where it gives a definite answer when it takes a known input pattern and gives approximate solution when it takes an unknown input pattern.

This research study showcases cutting-edge applications of AI methodologies in the fight against COVID-19, focusing on key enabling factors and limitations, and exploring future research directions. The subsequent sections delve into AI applications in COVID-19 control and management, as well as other related areas. Intelligent systems have been created that integrate risk assessment, decision support, and social sensing to support public health responses. Machine learning and deep learning models have expedited the discovery of anti-COVID-19 therapeutics through high-throughput screening. This study seeks to provide an overview of state-of-the-art AI techniques that are reinventing the battle against the COVID-19 pandemic. Using the power of AI, medical personnel can effectively combat the challenges posed by the current pandemic and pave the way for a healthier future. The availability of professional health information systems can make it easy to apply the AI model in diagnosis and remedy of any pandemic. These data infrastructures can be driven from the international health organizations and from health insurance companies.

### Baltimore classification

Classifying viruses according to their genome allows us to predict their behavior and guide future research. According to Baltimore classification, Viruses fall into seven main groups:

- I: dsDNA viruses (e.g. Adenoviruses, Herpesviruses, Poxviruses)
- II: ssDNA viruses (+) sense DNA (e.g. Parvoviruses)
- III: dsRNA viruses (e.g. Reoviruses)
- IV: (+) ssRNA viruses (+) sense RNA (e.g. Picornaviruses, Togaviruses)
- V: (−) ssRNA viruses (−) sense RNA (e.g. Orthomyxoviruses, Rhabdoviruses)
- VI: ssRNA-RT viruses (+) sense RNA with DNA intermediate in life-cycle (e.g. Retroviruses)
- VII: dsDNA-RT viruses (e.g. Hepadnaviruses)

**Figure 3:**
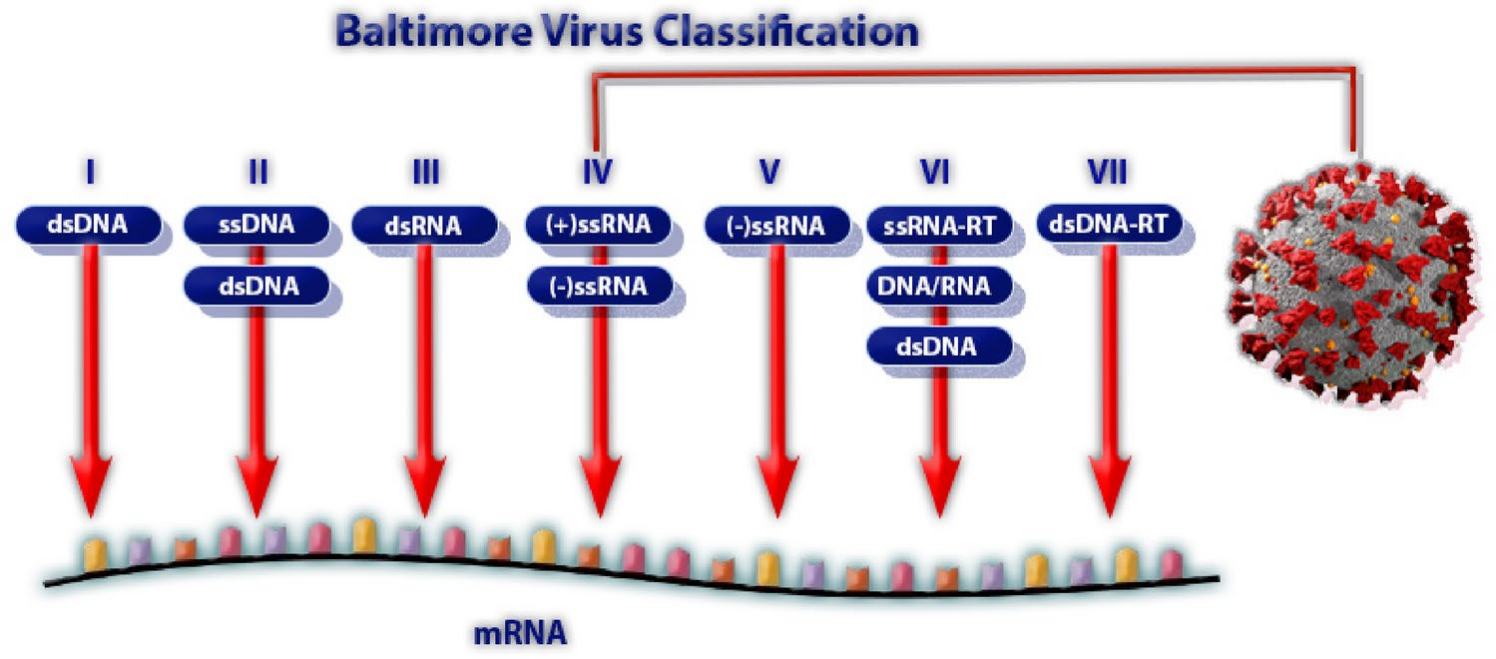
Diagnosis and remedy for COVID-19 based on Baltimore Virus Classification.

Virus corona, COVID-119, belong to the group four of Baltimore classification which is (+) sens single stranded RNA ((+)ssRNA). This group includes the rhinovirus (common cold). Commonly, it has fast mutation rate, fast wide-spreed infection, and mild symptoms except with cross-mortality diseases, see Fig. 3. From the fast mutation of group (IV) of Baltimore virus classification, we conclude that:

1. The innate immunity is more important than acquired immunity.
2. It is immunity disorder more than Acute Respiratory Disorder Stress(ARDS)

### Paper organization

The remainder of the paper is organized as follows, section “Survey of the related work” is the survey of the related works. Section “COVID-19 clinical symptoms and AI based diagnostics” introduces the AI based COVID-19 diagnosis. Section “COVID-19 AI augmented remedy” presents the AI based COVID-19 remedy. Finally, section “Conclusions” draws the conclusions.

## Survey of the related work

In Kosar et al.^1^ present a systematic review of COVID-19 diagnostic methods. They categorize techniques based on working principles and detection modalities, including chest radiography, cough sound analysis, RT-PCR, antigen testing, and antibody testing. A comparative assessment is conducted to assess their effectiveness, helping to identify the best solutions. The research emphasizes the important contribution of Artificial Intelligence in advancing COVID-19 diagnostic approaches to improve healthcare systems. This study provides a useful reference for researchers and presents durable techniques that can be applied to future pandemics.

In Shoeibi et al.^2^ provide a comprehensive review of DL techniques to diagnose COVID-19 from medical imaging. The article outlines diagnostic methods for coronavirus using medical imaging, highlighting the associated challenges. It delves into key aspects of Computer-Aided Diagnosis Systems (CADS) based on DL methods for COVID-19 diagnosis, covering segmentation, classification, explainable AI (XAI) and predictive research. Additionally, the review addresses rehabilitation systems like the Internet of Medical Things (IoMT) in the context of COVID-19. A separate section presents research on Uncertainty Quantification (UQ) in DL models for Covid-19 diagnosis. Critical challenges and future research directions are explored in another section, followed by discussion and conclusion sections to wrap up the paper.

In Gheisari et al.^3^ present a multidimensional systematic review of mobile apps for the detection and diagnosis of COVID-19 for future pandemic control.

In Farahat et al.^4^ present an AI-driven diagnostic system designed to predict the respiratory support needed for patients with COVID-19. The system analyzes the relationship between COVID-19 lesions and the required level of respiratory support. It utilizes computed tomography (CT) imaging to examine the three levels of support: Level 0 (minimal support), Level 1 (non-invasive support such as oxygen) and Level 2 (invasive support such as mechanical ventilation). Initially, the system segments COVID-19 lesions from CT images and creates a unique appearance model for each lesion using a 2D, rotation-invariant, Markov-Gibbs random field (MGRF) model. Three MGRF-based models are developed, one for each support level, enabling the system to distinguish between varying levels of severity in patients with COVID-19. A neural network-based fusion system is then employed to make decisions for each patient by combining the estimates of Gibbs energy from the three MGRF-based models.

In Fang et al.^5^ present a review aimed at providing insight into the diverse solutions created to tackle the complex challenges that arise during the pandemic. Their objective is to equip the AI community with the necessary knowledge to develop AI tools that can effectively address public health emergencies.

In^6^ The authors collected 2,400,200 CT slices from 12 emergency centers in Japan. They calculated the Area Under Curve (AUC) and precision to evaluate classification performance. The system’s inference time, incorporating these two models, was also recorded. In the validation data, the slice and series models identified COVID-19 with AUCs and accuracies of 0.989 and 0.982 (95.9% and 93.0%), respectively.

In^7^ The authors retrospectively evaluate these proposed studies and review the techniques used in AI diagnostic models, with a focus on solutions for different challenges. This review aims to provide information on solutions designed to address the multifaceted challenges that emerged during the pandemic. This will help prepare the AI community for the development of AI tools customized to effectively address public health emergencies.

The aforementioned studies indicate that the severity of COVID-19 can be accurately predicted using clinical and laboratory markers. The primary aim of this study is to anticipate the severity of COVID-19 in patients.

## COVID-19 clinical symptoms and AI based diagnostics

Clinical tests to assess the severity of COVID-19 usually involve evaluating symptoms, vital signs, blood tests, imaging studies, and other diagnostic tools. These tests help healthcare providers determine the level of illness and guide appropriate treatment interventions. Patients with COVID-19 exhibit a variety of symptoms. Fever is the most common (100%), while cough is present in 91% of the cases. Fatigue and myalgia affect between 5.9% to 75% and 3.33% to 70% of patients, respectively. Chest pain/tightness is also reported. Abdominal pain is documented and mental disorders and confusion occur at rates of 16.25% and 9%, respectively. Dizziness is less commonly reported in patients. Nasal congestion ranges from 61.5% to 6.9%. Other symptoms like shortness of breath (dyspnea), rhinorrhea, expectoration, chills, sore throat, nausea or vomiting, diarrhea, headache, and anorexia are less frequent.

**Figure 4:**
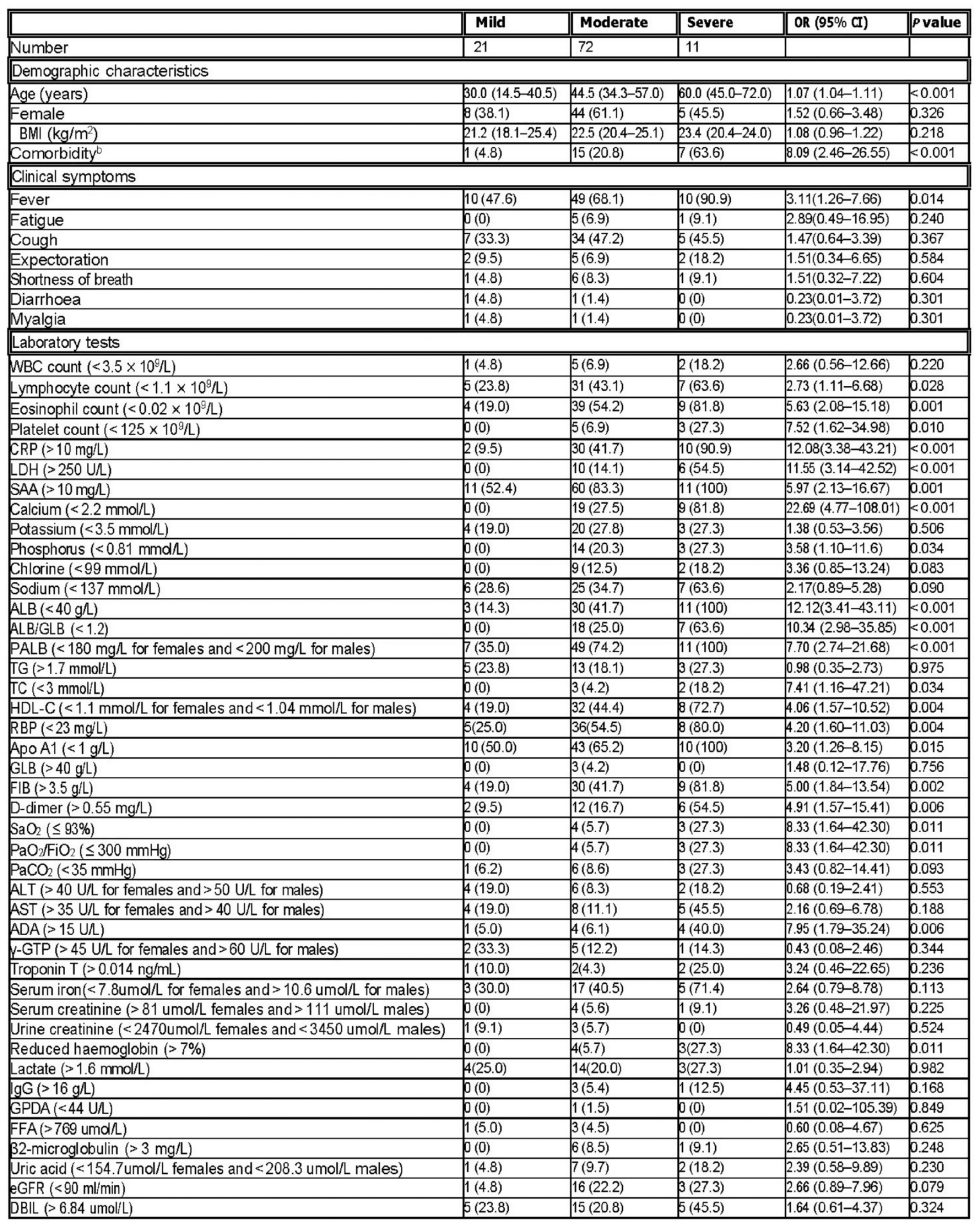
Clinical characteristics to the severity of COVID-19 training set^8^.

**Figure 5:**
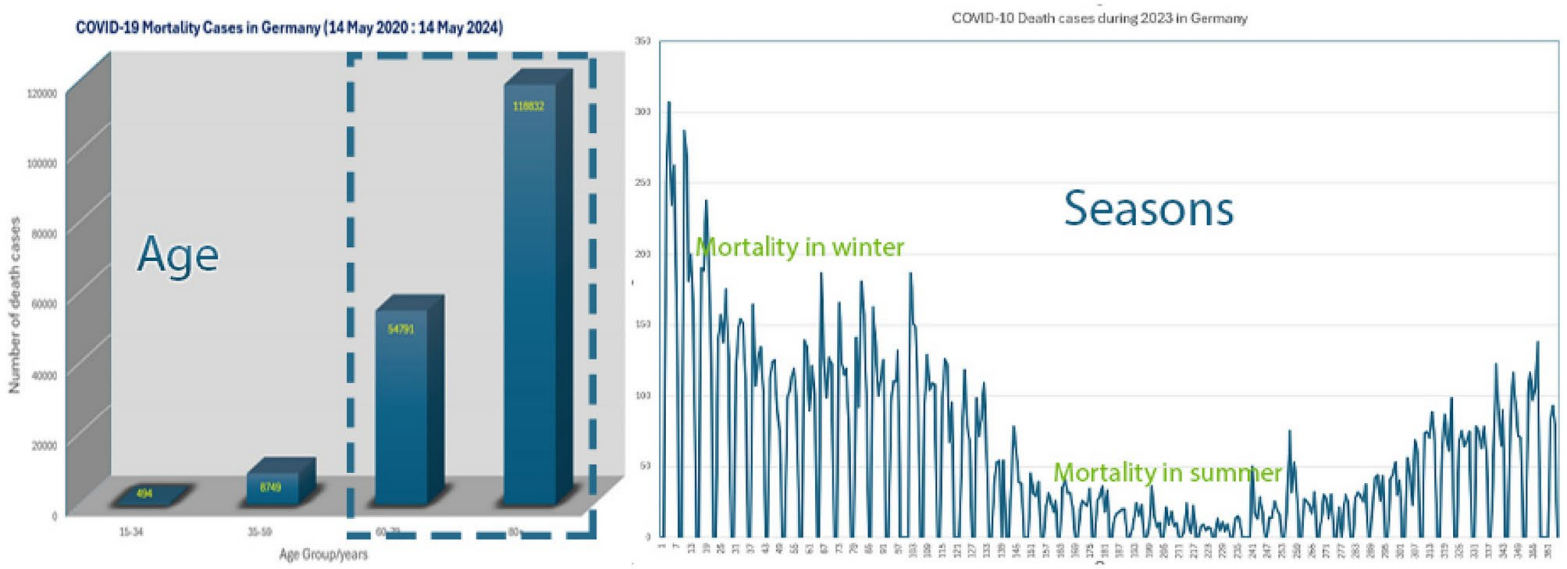
Mortality to Age and seasonal mortality in Germany.

The technique described in the Figs. 1 and 2 has been applied to age, comorbidity, fever, and 18 laboratory markers were associated with the severity of COVID-19 at Fig. 4^8^. The deep learning algorithm is shown in algorithm 1. The presented algorithm has been applied on NVIDIA Quadro T1000 supporting Max-Q Design (4 GB GDDR6 dedicated) GPU. The data set used a case study downloaded from (COVID-19-Todesfälle in Deutschland)) dataset. From this data set^9^, shown in Fig. 5, we discover that:

(3) The cross mortality due to aging is more dangerous than COVID-19.
(4) COVID-19 is a seasonal disease due to the deficiency of vitamin D in winter.
(5) COVID-19 is epidemic, not pandemic.

### COVID-19 pathogenic proinflammatory cytokine storm

Cohort studies have shown that elevated levels of circulating pro-inflammatory cytokines and chemokines are strongly linked to disease severity and mortality, with their underlying causes discussed extensively in other reviews. In particular, elevated levels of IL-6, IL-2, IL-7, IL-10, granulocyte colony stimulating factor (G-CSF), IP-10, MCP1, IFNy, macrophage inflammatory protein 1 $\alpha$ (MIP1 $\alpha$) and tumor necrosis factor (TNF) have been implicated in the severity of COVID-19, indicating a combined response of T-helper type 1 (Th1) and Th2 cells. Specifically, IL-6 has emerged as a potential treatment target due to its strong association with disease progression. A recent meta-analysis suggested serum IL-6 cut-offs of $>55$ pg/ml and $>80\,\text{pg}/\text{ml}$ to identify patients at high risk for severe COVID-19 and mortality, respectively. Prospective validation of these proposed cut-offs across different assay methodologies and patient populations is urgently awaited to establish clinical utility. Importantly, the cytokine concentrations observed in hospitalized COVID-19 patients are rarely as elevated as in secondary hemophagocytic lymphohistiocytosis and cytokine release syndrome following CAR-T cell treatment. This is the reason why I consider COVID-19 an immunity disorder more than a respiratory disease.

In immunity disorder, patients reach the COVID-19 severity leading to cytokine storm and elevated IL 6 as an inflammation marker for severe COVID-19 infection with poor prognosis and this proves that COVID-19 is an immunological disorder. The elevation of IL 6 in severe COVID-19 which causes the cytokine storm indicates that the immunity disorder is the main reason behind health deterioration, not COVID-19. High levels of several key pro-inflammatory cytokines, such as IL-1, IL-2, IL-6, TNF-$\alpha$, IFN-$\gamma$, IP-10, GM-CSF, MCP-1, and IL-10, some of which also correlate with COVID-19 severity. COVID-19 is mainly immunity disorder.

### Molecular diagnosis

Molecular diagnosis is a crucial aspect of modern medicine, with nucleic acid testing its key technology. Nucleic acid testing is vital for precise coronavirus identification due to its ability to pinpoint specific pathogens. Current nucleic acid detection methods include gene sequencing, CRISPR, and nucleic acid amplification tests such as PCR or isothermal nucleic acid amplification. PCR, known for its thermal cycling requirement, is highly sensitive and specific in detecting viruses. Isothermal nucleic acid amplification, on the other hand, offers rapid detection under constant temperature without the need for a thermocycler. By March 24, 2020, the genome and proteome compositions of the virus had been elucidated, although the host’s response to SARS-CoV-2 remained incompletely understood. More than 1000 COVID-19 sequences have been shared with the public to date.

The Reverse transcriptase-polymerase chain reaction cycle threshold (RT-PCR Ct) value refers to the number of cycles needed to replicate enough DNA/RNA to be detected (crosses a threshold line). The lower Ct value means there was more DNA/RNA in the sample to begin with. The severity indications in CXR and CT lowers the Ct value.

#### PCR-based testing methods

PCR tests utilize DNA amplification technology with the enzymatic activity of a DNA polymerase to multiply the desired gene fragment. Detecting coronavirus involves a reverse transcription step converting viral RNA into cDNA, followed by PCR amplification for quantitative detection of the fluorescent reaction using specific instruments.

#### Isothermal nucleic acid amplification techniques (INAATs)

INAATs are innovative alternatives to traditional PCR methods. These techniques allow for exponential amplification of nucleic acids at constant temperatures, eliminating the need for temperature cycling. Let us dive into some key aspects of INAATs. The isothermal amplification methods are Nucleic acid sequence-based amplification (NASBA), Loop-mediated isothermal amplification (LAMP), Helicase-dependent amplification (HDA), Rolling circle amplification (RCA), Multiple displacement amplification (MDA), whole genome amplification (WGA), Recombinase polymerase amplification (RPA).

For COVID-19, the next two techniques are important.

1. *Loop-Mediated Isothermal Amplification (LAMP)*: LAMP is a fast and simple technique that works under isothermal conditions. It employs Bacillus stearothermophilus DNA polymerase (Bst DNA polymerase). The method includes a strand displacement reaction and the creation of stem-loop structures. Primers specially designed bind to various regions of the target DNA sequence. LAMP has been used in point-of-care (POC), laboratory, and field tests, such as SARS-CoV-2 detection during the COVID-19 pandemic.
2. *Multiple Displacement Amplification (MDA)*: MDA is a commonly used whole genome amplification (WGA) method. It employs a strand-displacing DNA polymerase, like Phi29 polymerase, in conjunction with random hexamers. The genome is amplified under isothermal conditions.

These isothermal techniques are crucial for disease detection, research, and diagnostic applications. Their simplicity, speed, and suitability for point-of-care testing make them valuable tools in the field of molecular biology.

### AI based COVID-19 medical image processing

The formal diagnosis of COVID-19 necessitates a laboratory test (RT-PCR) on samples collected from the nose and throat. RT-PCR testing calls for specialized equipment and typically yields results in a minimum of 24 hours. Chest imaging has proven crucial in understanding the progression of this respiratory illness. A swift and precise diagnosis of COVID-19 can be achieved through the analysis of chest radiographs (CXR) and computed tomography (CT) images. This study seeks to evaluate the efficacy of different chest imaging methods in diagnosing COVID-19 with the assistance of AI. CT scans demonstrate a 5% higher accuracy compared to CXR results^10,11^.

GGOs show up as lighter-colored or gray patches on chest CT scans of the lungs. This finding can indicate COVID-related high severity lung conditions, such as pneumonia. Other causes of GGOs include congestive heart failure, diffuse alveolar hemorrhage, interstitial lung diseases, and lung cancer. For sure the GGOs is clear in CT compared with CXR.

#### CT versus CXR

**Figure 6:**
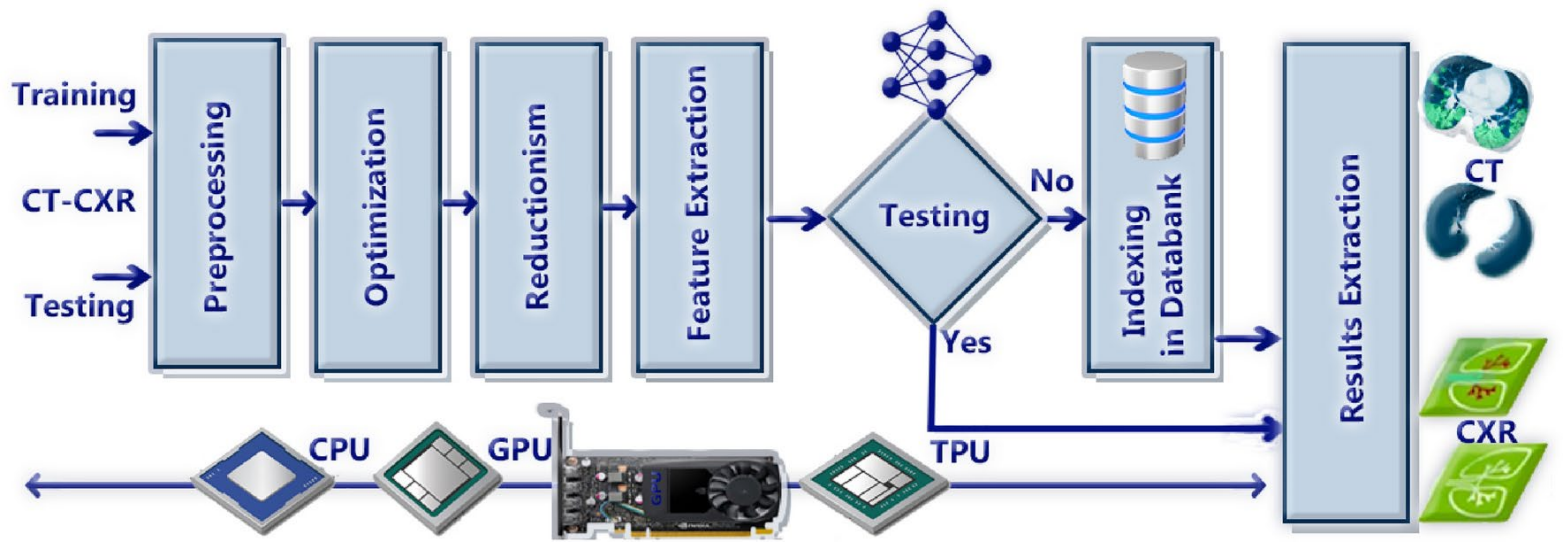
CT-CXR image processing workflow.

Chest CT scans offer a more detailed view than chest X-rays. While an X-ray can detect abnormalities, a CT scan can pinpoint the exact location and analyze the nature of a formation. X-rays provide a 2D image, whereas CT scans generate a 3D view of your organs. X-rays are designed to examine dense tissues, whereas CT scans excel at capturing bones, soft tissues, and blood vessels simultaneously. CT scanners are larger and more complex than X-ray machines, since they need to rotate around the patient during scanning. A chest X-ray is a cost-effective initial examination, but a chest CT scan may be necessary for a comprehensive diagnosis and treatment plan. The image processing process has two phases, the first is the training and the second is the testing. Both pass through preprocessing, optimization, reductionism, and feature extraction. In the training phase, the data are indexed and stored in data bank while testing extracts the results, see Fig. 6.

The power of approximation is the most important feature of DL where it gives a definite answer when it takes a known input patterns and gives approximate solution when it takes unknown input pattern. The programming is done by Python, Keras, and Tensorflow Deep Learning (DL) using GPU NVIDIA Quadro T1000 with Max-Q Design HP ZBook Power G7 Mobile Workstation, Core i9, see algorithm 1. The precision can be estimated as 97% and recall as 70% with 85% F1 score.

#### Image processing

1. Datasets

In the medical field, there are various image screening technologies, such as ultrasound, CT scans, MRI, and X-ray imaging. Radiologists utilize these images to diagnose organ conditions and detect abnormalities. Disease detection through CXR images poses challenges for radiologists, occasionally resulting in misdiagnoses. To improve accuracy, CAD systems necessitate extensive data for training and testing. In medical analysis, CAD systems are typically trained and tested on datasets comprising images and metadata (e.g., patient age, race, sex, insurance type). Institutions around the world adopt diverse approaches to collect patient data for research purposes. The collection of data sets in medicine aims to enhance disease detection research. Deep learning techniques have shown their efficacy in detecting severe illnesses in various datasets, achieving expert-level performance in clinical tasks. Numerous datasets encompass thousands of CXR images. In this research work, four datasets have been used to detect the severity of COVID-19, they are 1. COVID-19 Lung CT Scans, 2. COVID-19 Radiography Database, 3. COVID-19-Todesfälle in Deutschland, and 4. Our world in data.

2. Preprocessing

Medical image preprocessing involves enhancing the quality of images by converting them into a more usable and informative format from their original form. DICOM (Digital Imaging and Communications in Medicine) is the common format for medical images, often with extensive metadata that can be challenging for non-radiology experts to interpret. In fields like computer vision, DICOM images are typically converted to PNG or JPG formats using specific compression algorithms to maintain important information. This process involves removing private patient data for privacy protection and converting DICOM images to PNG, JPEG, or other formats. Due to their high dimensions, radiological images often need to be resized to reduce computational resources while preserving essential information. Preprocessing commonly includes denoising, normalization, artifact/bone removal, enhancement, and segmentation of images in medical datasets^12,13^.

3. Deep Learning

The power of approximation is the most important feature of DL where it gives a definite answer when it takes a known input pattern and gives approximate solution when it takes an unknown input pattern.

**Figure 7:**
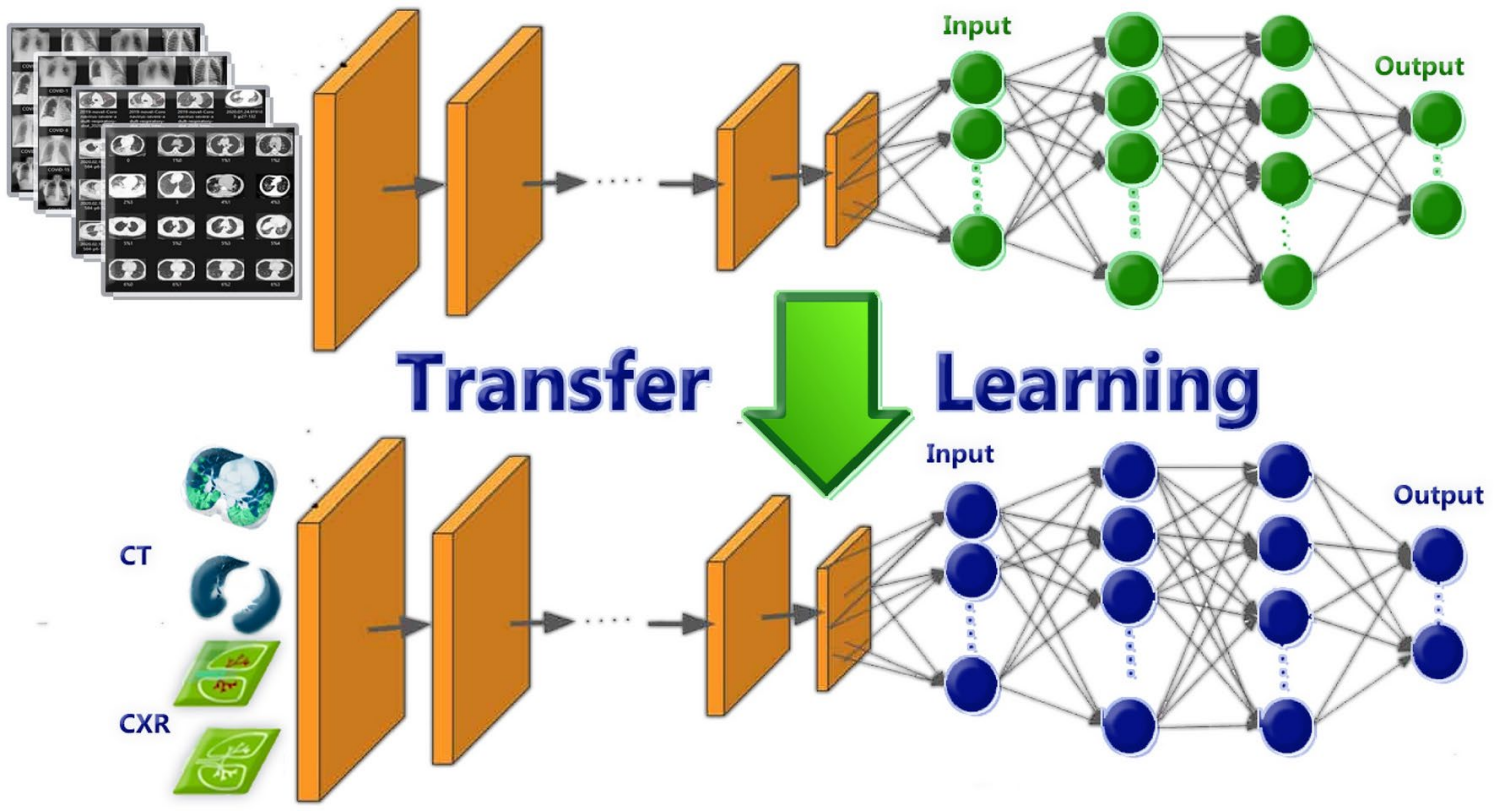
Applying transfer learning to improve DL results.

Transfer learning in deep learning (DL) involves applying the knowledge gained from a previously trained model to a related problem. For example, a model trained to identify COVID-19 positive images can leverage its learned features to recognize other objects. The core idea is to utilize insights gained from a well-labeled dataset to enhance performance on a new task with limited data, rather than starting from scratch. Transfer learning is commonly used in computer vision, medical data processing, and natural language processing tasks like sentiment analysis, primarily due to the significant computational resources involved. While not exclusive to DL, it is often combined with neural networks that require extensive data and processing power. This approach not only improves the efficiency of training DL models but also reduces the time and resources needed for development. By fine-tuning pre-trained models on smaller datasets, practitioners can achieve competitive results even in data-scarce situations, which is particularly beneficial in fields like medical imaging, where obtaining labeled data can be costly and time-consuming.

**Algorithm 1:**
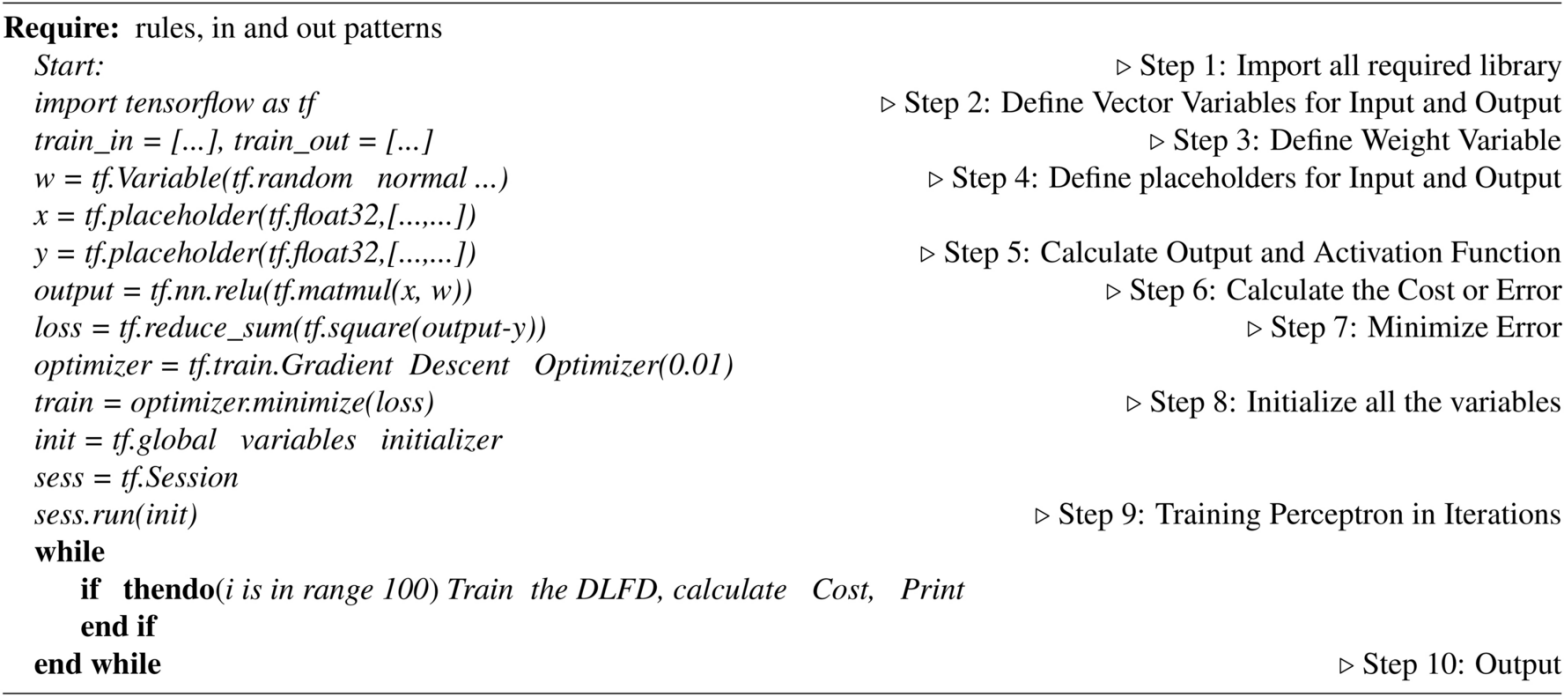
Deep Learning based COVID-19 Diagnosis algorithm^14^

 In practice, transfer learning typically involves two main phases: pre-training and fine-tuning. During the pre-training phase, a model is trained on a large, diverse dataset, allowing it to learn general features that are applicable across various patterns. For instance, a GL trained on a vast collection of medical images can learn to identify edges, textures, and shapes. In the fine-tuning phase, this pre-trained model is adapted to a specific task by retraining it on a smaller, task-specific dataset. This process often involves modifying the final layers of the model to better suit the new classification problem, while freezing the earlier layers to retain the learned features. Moreover, the success of transfer learning is also attributed to the hierarchical nature of deep learning models. Early layers typically capture low-level features, while deeper layers represent more abstract concepts. This hierarchy allows for a seamless transfer of knowledge, as the foundational features learned from one group of patterns can be beneficial for another, even if the latter is quite different in nature. Transfer learning has also paved the way for more innovative approaches, such as few pattern learning, where models are designed to generalize from very few patterns or even to recognize entirely new classes without any prior learning. These advancements highlight the versatility and power of transfer learning in pushing the boundaries of what is achievable in DL. Transfer learning stands as a cornerstone of modern DL practices, enabling researchers and practitioners to build robust models efficiently. Its ability to harness previously acquired knowledge not only accelerates the development process but also opens up new possibilities for tackling complex problems across various applications. As the field continues to evolve, the integration of transfer learning with emerging techniques will likely lead to even more groundbreaking advancements in AI, see Fig. 7.

**Figure 8:**
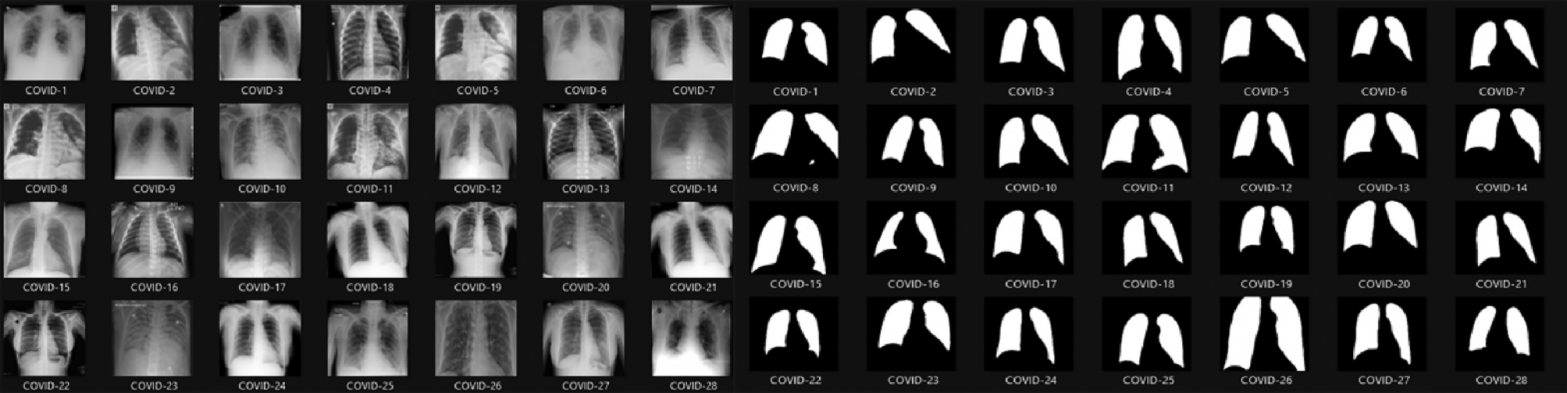
COVID-19 diagnosis in 3616 CXR medical imagery positive cases with 92% accuracy.

**Figure 9:**
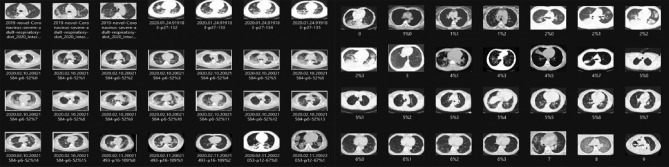
349 positive COVID-19 diagnosis CT and 397 negative medical imagery with 97% accuracy.

Several CAD systems have been developed for detecting chest diseases using various techniques. Early diagnosis of thoracic conditions provides an opportunity to combat the disease effectively. Diseases such as tuberculosis, pneumonia, and COVID-19 pose greater risks and become more severe as they progress. In medical imagery, three primary types of abnormalities are typically observed: (1) texture abnormalities, characterized by diffuse changes in the appearance and structure of the area. (2) Focal abnormalities, manifesting as isolated density changes, and (3) Abnormal forms that alter the outline of normal morphology. This paper presents a myriad of models and systems designed to address chest diseases using deep learning (DL) methodologies. The focus is on the DL algorithms utilized for detecting various chest ailments using medical CT/CXR images. The DL algorithm is described in algorithm 1. The results of the COVID-19 diagnosis 28 cases from 3616 CXR medical imaging positive cases with 92% precision are presented in Fig. 8. The results of 349 positive and 397 negative CT medical images for the precision diagnosis of COVID-19 97% are presented in Fig. 9.

4. Adam optimization

Stochastic gradient-based optimization is crucial in various scientific and engineering fields. Many problems can be formulated as optimizing a scalar objective function by maximizing or minimizing it with respect to its parameters. When the function is differentiable with respect to these parameters, gradient ascent provides a relatively efficient optimization method, as computing first-order partial derivatives has the same computational complexity as evaluating the function itself. Frequently, the objective functions encountered are stochastic.

*Adam optimization* is an adaptive learning rate algorithm for training deep learning models. It combines the benefits of two extensions of stochastic gradient descent: AdaGrad and RMSProp. By computing unique adaptive learning rates for various parameters based on first and second moment estimates of gradients, Adam is efficient for large-scale data and high-dimensional problems. It offers a straightforward implementation, requires minimal memory, and is suitable for large datasets and parameter spaces, see algorithm 2.

**Algorithm 2:**
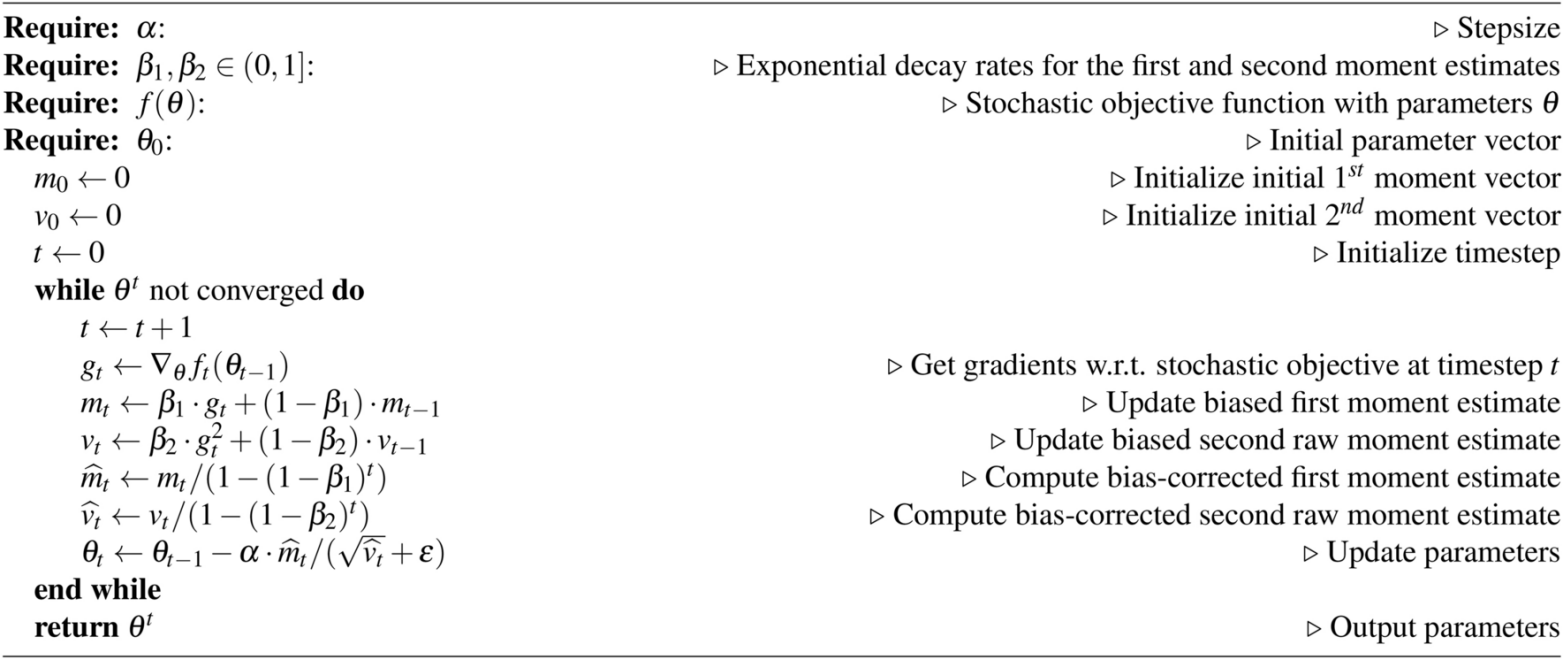
*Adam algorithm* for DL optimization. $g^2_t$ indicates the elementwise square $g_t \odot g_t$. Good default settings for DL problems in these experiments were $\alpha =0.0002$, $\beta _1=0.1$, $\beta _2=0.001$ and $\epsilon = 10^{-8}$.

## COVID-19 AI augmented remedy

Some of COVID-19 symptoms are; Fever, dry cough, headache, dizziness, rhino-rhea, myalgia, chills, rigors, diarrhea, vomiting. Lower respiratory tract infection (LRTI), acute respiratory distress syndrome (ARDS), shock, and multiorgan failure.

**Figure 10:**
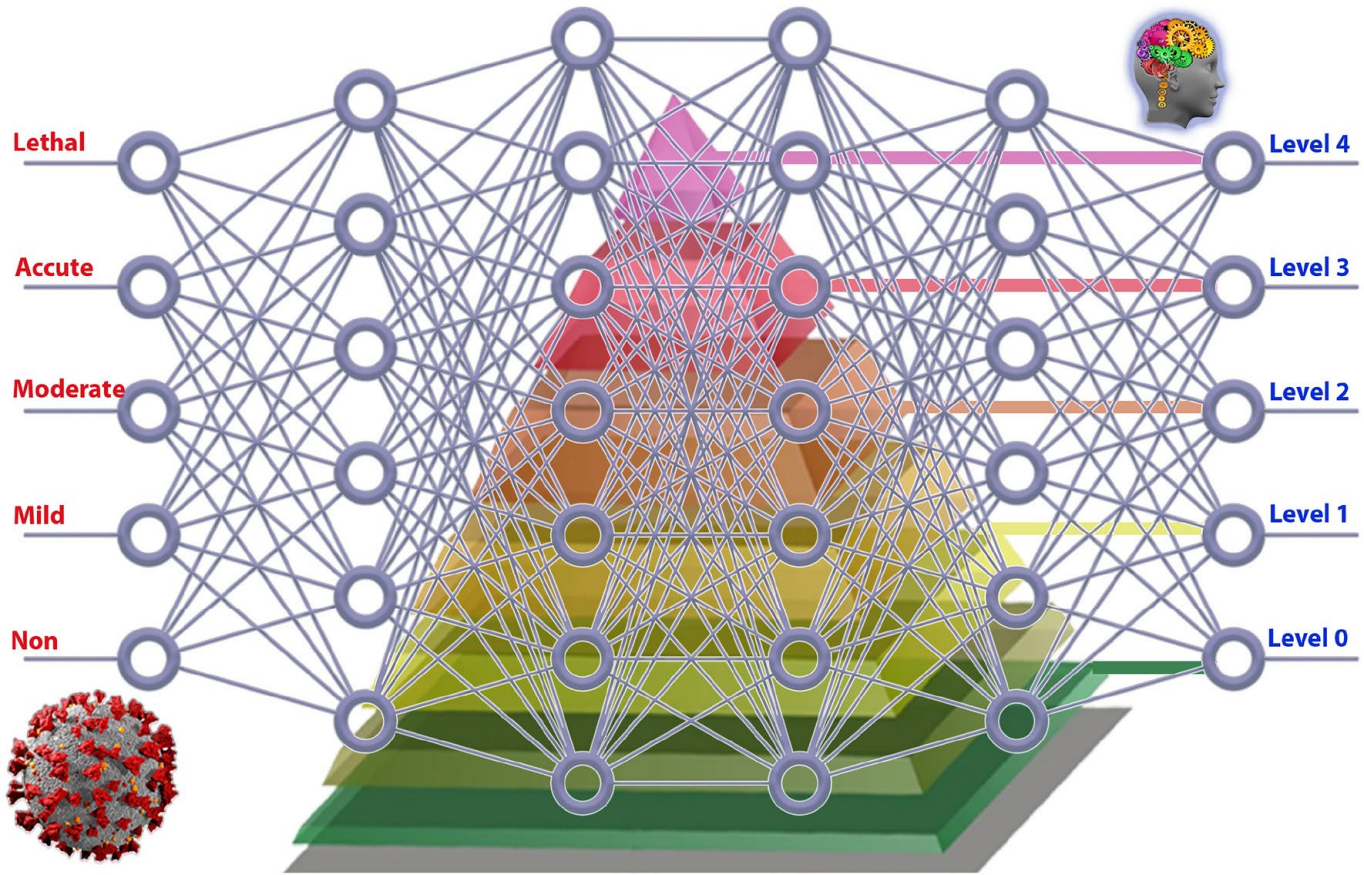
DL implementation of COVID-19 AI based Remedy.

- *Mild*: Patients with mild illness may show various signs and symptoms (e.g., fever, cough, sore throat, malaise, headache, muscle pain, nausea, vomiting, diarrhea, loss of taste and smell). They do not experience shortness of breath, dyspnea in exertion, or abnormal imaging. Most mildly ill patients can be treated in an ambulatory setting or at home. Imaging or specific lab tests are not typically needed for otherwise healthy patients with mild COVID-19. Patients aged $\ge$ 50 years, especially those aged $\ge$ 65 years, patients with certain underlying comorbidities, and patients who are immunosuppressed, not vaccinated, or not up-to-date with COVID-19 vaccinations are at increased risk of disease progression and are candidates for antiviral therapy.
- *Moderate*: Moderate illness is defined as evidence of lower respiratory disease during clinical assessment or imaging, with an SpO$_2$ $\ge$ 94% on room air at sea level. Given that pulmonary disease can progress rapidly in patients with COVID-19, patients with moderate disease should be closely monitored.
- *Accute*: Patients with COVID-19 are considered to have a serious illness if they have an SpO$_2$ $\le$ 94% on room air at sea level, PaO$_2$/FiO$_2$ $\le$ 300 mm Hg, a respiratory rate $\ge$ 30 breaths / min or infiltrates of the lung $\ge$ 50%. These patients may experience rapid clinical deterioration and should be given oxygen therapy and hospitalized.
- *Lethal*: SARS-CoV-2 infection can cause acute respiratory distress syndrome, virus-induced distributive shock (septic), cardiac shock, an exaggerated inflammatory response, thrombotic disease, and exacerbation of underlying comorbidities.

**Figure 11:**
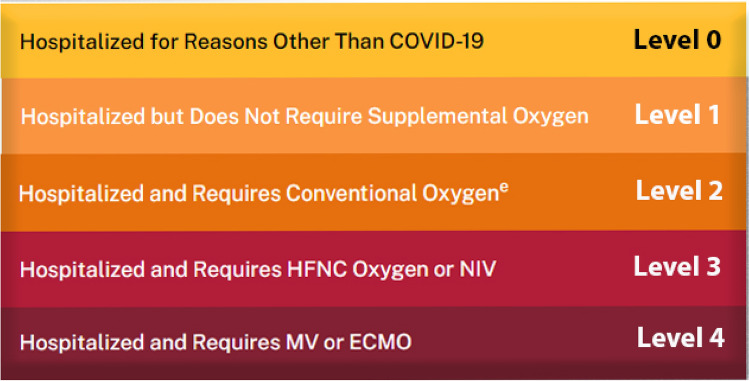
Hospitalization according to COVID-19 severity.

According to the severity, AI can determine the appropriate remedy. The DL algorithm found in Algorithm 1 applied on the ANN found in Fig. 10, will lead to get the results in Fig. 11.

**Figure 12:**
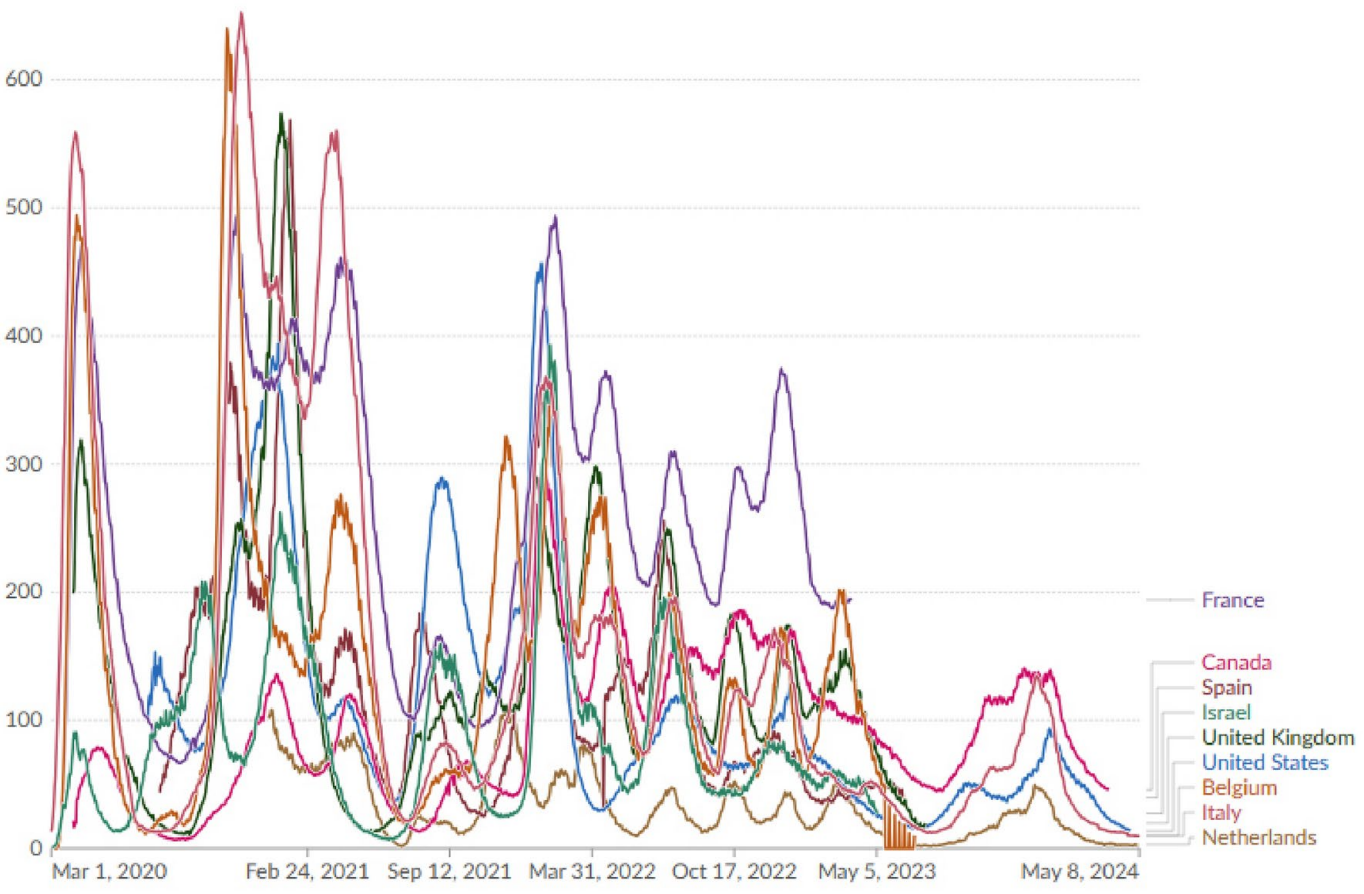
Number of COVID-19 patients in hospital per million in 9 countries.

1. *Supplemental Micronutrients*: Certainly! Although micronutrient supplements cannot replace vaccination or other preventive measures, they may help support your immune system. Here are some micronutrients that have been studied in relation to COVID-19:
  *Vitamin D*: Vitamin D has immunomodulatory effects and can help reduce the severity of respiratory diseases. Some research indicates that maintaining sufficient levels of vitamin D could be advantageous for individuals with COVID-19.
  *Vitamin C*: Vitamin C is renowned for its antioxidant properties and its ability to bolster immune function. Reduced serum vitamin C levels have been observed in critically ill patients with COVID-19.
  *Zinc*: Zinc is crucial for immune function and has been researched for its antiviral properties. It could potentially lessen the duration and intensity of viral infections, such as COVID-19.
  *Selenium*: Selenium plays a role in antimicrobial activity and inflammation reduction.
  It is important to remember that supplements should not replace a healthy diet, but can complement it.
2. *Natural Disinfectants*: several natural disinfectants should be discussed, such as;
  *Natural Production of Nitric Oxide*: Nitric oxide (NO) plays a vital role in the context of COVID-19. NO is a signaling molecule naturally produced by the body, particularly in the endothelial cells lining our arteries and veins, with a significant presence in the lungs. Endothelium-derived NO aids in blood vessel relaxation, reducing high blood pressure and facilitating blood flow to all organs. Moreover, NO prevents blood clots in healthy arteries and relaxes smooth muscle in the airways, easing breathing. It also exhibits direct antiviral properties. In a study during the SARS outbreak, NO-releasing compounds increased the survival rate of mammalian cells infected with SARS-CoV, hinting at NO’s potential to hinder coronavirus replication, including SARS-CoV-2, the virus responsible for COVID-19.
  *Hydrogen peroxide*: Hydrogen peroxide is commonly used as a disinfectant and can effectively kill COVID-19 on surfaces. When appropriately diluted, it can be useful for cleaning and sanitizing frequently touched objects and surfaces. Some social media claims suggest inhaling hydrogen peroxide through a nebulizer can prevent or cure COVID-19, but this is not supported by scientific evidence. The Asthma and Allergy Foundation of America warns against inhaling hydrogen peroxide, as it can damage the lungs and is potentially dangerous. A 2012 study found that inhaling highly concentrated solutions of hydrogen peroxide can cause severe irritation and inflammation of mucous membranes.
  *Glutathione*: Glutathione is the most abundant physiological antioxidant, has garnered attention in the context of COVID-19. Researchers at Baylor College of Medicine investigated the impact of COVID-19 on oxidative stress, oxidant damage, and glutathione levels. Increased oxidative stress and oxidant damage Markedly reduced levels of glutathione1. The study suggests that supplementation with GlyNAC, a combination of glutathione precursors, might benefit COVID-19 patients. GlyNAC has been shown to reduce oxidative stress and oxidant damage and increase glutathione levels. It improves health indicators like inflammation. However, GlyNAC’s specific association with COVID-19 remains unstudied.
  *Methylene blue*: Methylene blue has been studied in relation to COVID-19. Researchers have found that methylene blue inhibits the interaction between the SARS-CoV-2 spike protein and its receptor ACE2. This interaction is crucial for viral attachment and entry into cells. In laboratory experiments, methylene blue demonstrated inhibitory activity against this protein-protein interaction, with a low micromolar half-maximal inhibitory concentration (IC50) of 3 $\upmu M$. Chloroquine, siramesine, and suramin did not show similar inhibitory effects. Additionally, methylene blue inhibited the entry of SARS-CoV-2 spike-bearing pseudovirus into ACE2-expressing cells. This PPI inhibitory activity suggests that methylene blue could potentially have antiviral effects against SARS-CoV-2, even without light activation. It might be useful as an oral or inhaled medication for COVID-19 treatment.
  *UVC Light and SARS-CoV-2 Inactivation*: UVC light is the most effective type of UV light for killing germs, including viruses and bacteria. It damages molecules like nucleic acids and proteins, rendering the germ incapable of survival. Recent studies have investigated UVC light’s effectiveness against SARS-CoV-2, the novel coronavirus responsible for COVID-19. A study in the American Journal of Infection Control found that UVC light exposure completely inactivated the virus in liquid cultures within 9 minutes. Another study in the same journal used a specific type of UVC light called far-UVC light (wavelengths between 207 and 222 nanometers). This far-UVC light reduced live coronavirus on laboratory surfaces by 99.7% in just 30 seconds. A study published in Scientific Reports explored using far-UVC light to kill two other human coronaviruses (229E and OC43) in the air. While not directly tested on SARS-CoV-2, this study suggests potential efficacy.
3. *Anti-Inflamation*: There are several medications that have been studied for their potential effectiveness such as Nirmatrelvir-Ritonavir (Paxlovid), Remdesivir (Veklury), Molnupiravir, Baricitinib, and Dexamethasone.
4. *ICU+ and Artificial Organs*: The strain on intensive care units (ICUs) has been immense, leading to shortages of critical resources such as ventilators, Direct Peritoneal Resuscitation, artificial Kidney, and ECMO.
5. *Hospitalization*: Anyone has the potential to contract COVID-19 and experience severe illness or mortality, although the majority will recuperate spontaneously, devoid of medical intervention. Individuals aged 60 and above, as well as those with preexisting health issues, are at an elevated susceptibility for developing severe symptoms. These underlying conditions encompass hypertension, diabetes, obesity, immunosuppression such as HIV, cancer, and pregnancy. Additionally, individuals who have not been vaccinated are also at a heightened risk of encountering severe manifestations. The statistics of CONVID-19 hospitalization in 9 countries are shown in Fig. 12, for more read our world in data.

## Conclusions

Throughout this article COVID-19, caused by the SARS-CoV-2 coronavirus, has spread to more than 200 countries, has affected millions, cost billions, and has claimed nearly 2 million lives since late 2019. This highly contagious disease can easily overwhelm healthcare systems if not managed promptly. The current diagnostic method, Molecular diagnosis, is slow and has low sensitivity. CXR, an initial imaging tool, provides rapid results, but is less sensitive compared to CT scans. This article focuses on using AI for two main objectives: classifying the severity of COVID-19 and determining the appropriate treatment. This research highlights key factors in the diagnosis and management of COVID-19, addressing answers for the questions asked in the abstract of this paper as follows: 1. For COVID-19, innate immunity is more important than acquired immunity. 2. COVID-19 is an immunity disorder more than Acute Respiratory Distress Syndrome (ARDS). 3. Cross-morbidity due to aging and autoimmune diseases or organ failure is more dangerous than COVID-19. 4. COVID-19 is a seasonal disease due to the deficiency of vitamin D in winter. 5. It is better to deal with COVID-19 as epidemic, more than pandemic.

## Data Availability

The data used in this research are available under: 1. https://www.kaggle.com/datasets/luisblanche/covidct. 2. https://www.kaggle.com/datasets/tawsifurrahman/covid19-radiography-database. 3. https://zenodo.org/records/10969632. 4. https://ourworldindata.org/grapher/current-covid-hospitalizations-per-million. For more information on the code and data, please, contact the corresponding author: Ashraf Aboshosha, Email: ashraf.aboshosha@eaea.sci.eg
